# FUNDC1: An Emerging Mitochondrial and MAMs Protein for Mitochondrial Quality Control in Heart Diseases

**DOI:** 10.3390/ijms24119151

**Published:** 2023-05-23

**Authors:** Xizhe Bai, Zhe Zhang, Xi Li, Yangjun Yang, Shuzhe Ding

**Affiliations:** 1 Key Laboratory of Adolescent Health Assessment and Exercise Intervention, Ministry of Education, East China Normal University, Shanghai 200241, China; 51251000030@stu.ecnu.edu.cn (X.B.); zhangzhe@tyxx.ecnu.edu.cn (Z.Z.); LiXi989508060506@163.com (X.L.); yjyang98@163.com (Y.Y.); 2 College of Physical Education and Health, East China Normal University, Shanghai 200241, China

**Keywords:** HDs, MQCs, FUNDC1, MAMs, exercise

## Abstract

Heart diseases (HDs) are the leading cause of mortality worldwide, with mitochondrial dysfunction being a significant factor in their development. The recently discovered mitophagy receptor, FUNDC1, plays a critical role in regulating the homeostasis of the Mitochondrial Quality Control (MQC) system and contributing to HDs. The phosphorylation of specific regions of FUNDC1 and varying levels of its expression have been shown to have diverse effects on cardiac injury. This review presents a comprehensive consolidation and summary of the latest evidence regarding the role of FUNDC1 in the MQC system. The review elucidates the association of FUNDC1 with prevalent HDs, such as metabolic cardiomyopathy (MCM), cardiac remodeling/heart failure, and myocardial ischemia-reperfusion (IR) injury. The results indicate that the expression of FUNDC1 is elevated in MCM but reduced in instances of cardiac remodeling, heart failure, and myocardial IR injury, with divergent impacts on mitochondrial function among distinct HDs. Exercise has been identified as a powerful preventive and therapeutic approach for managing HDs. Additionally, it has been suggested that exercise-induced enhancement of cardiac function may be attributed to the AMPK/FUNDC1 pathway.

## 1. Introduction

The emergence of our collective understanding of mitochondria can be traced from the initial concept of “bioblasts [1]” to the development of adenosine triphosphate (ATP) synthesizing chemiosmotic machines [2], which ultimately contributed to the establishment of the “powerhouse of the cell” analogy. Following this, studies have demonstrated that mitochondria possess their own genome, known as mitochondrial DNA (mtDNA) [3], and that mutations in mtDNA can lead to various diseases [4]. This has piqued the interest of researchers in the field of mitochondria and its implications for human health. Mitochondria have been found to play a crucial role in numerous fundamental biological and signal transduction mechanisms, such as programmed cell death [5], calcium regulation [6], inflammatory responses [7], cellular metabolism [8], and oxidative stress [9], according to recent research. Furthermore, the allocation of mitochondria is contingent upon energy availability, with cardiac tissue exhibiting a high concentration of these organelles owing to the heart’s elevated metabolic demands and rapid energy turnover [10]. Given the abundance of mitochondria in cardiomyocytes, it is unsurprising that dysfunction of these organelles can significantly impact cellular activity, contribute to cardiac damage, and ultimately promote the development of heart diseases (HDs). The impairment of mitochondria results in a decrease in the production of ATP and the discharge of a sequence of stress-inducing molecules through the mitochondrial permeability transition pores (mPTP), such as mitochondrial DNA (mtDNA), reactive oxygen species (ROS), and cytochrome C. These factors contribute to the apoptosis of cardiomyocytes and exacerbate HDs [11]. Therefore, the maintenance of mitochondrial homeostasis is of utmost significance for the preservation of cardiomyocyte health. The Mitochondrial Quality Control (MQC) system is a well-conceived and effective mechanism utilized by cells to protect against mitochondrial damage-induced cell death. This system encompasses three distinct yet well-coordinated mechanisms: mitochondrial biogenesis, dynamic, and mitophagy.

The discovery of FUNDC1 as a mitophagy receptor was initially made by Professor Quan Chen and his team [12]. It resides in the outer mitochondrial membrane (OMM) [12] and mitochondrial-endoplasmic reticulum (ER) associated membranes (MAMs) [13]. Similar to other mitophagy receptors, FUNDC1 contains an LC3 interaction region (LIR) motif that binds with LC3 [12], resulting in mitophagosome expansion and evacuation of damaged mitochondria. The prompt elimination of impaired mitochondria would effectively impede the progression of cell death. In contrast to mitophagy, mitochondrial biogenesis encompasses the processes of generating new mitochondria, augmenting mitochondrial content, rejuvenating existing mitochondria, and elevating mitochondrial oxidative phosphorylation (OxPhos) capacity. Mitochondrial dynamics encompass both fission and fusion, which enable inter-mitochondrial interactions. Multiple studies have demonstrated that the deficiency of FUNDC1 led to the reformation of mitochondrial biogenesis and dynamic balance [13,14], as well as a decrease in the formation of MAMs due to FUNDC1 ablation [15,16]. These findings highlight the crucial role of FUNDC1 in regulating mitochondrial function. MAMs serve as the connection between the ER and mitochondria. MAMs provides an effective scaffold for the crosstalk between ER and mitochondria, playing a pivotal role in various signaling pathways that enables the rapid exchange of biological molecules to maintain cellular health [17]. Reports indicate that dysfunctional MAMs are associated with a range of pathological conditions and human diseases, such as neurodegenerative disorder [18], metabolic syndrome [19], and HDs [20].

Based on the above premises, it is posited that FUNDC1 may serve as the mediator of the MQC system, facilitating the coordination of three discrete MQC processes. Consequently, the primary objective of this review is to explicate the present state of FUNDC1 in mitochondrial biogenesis, dynamics, and mitophagy, along with the mechanism of the FUNDC1-mediated MQC system in diverse HDs, including metabolic cardiomyopathy, cardiac remodeling/heart failure, and myocardial ischemia-reperfusion injury. Ultimately, we suggest a novel mechanism whereby exercise may enhance cardiac function through the FUNDC1-mediated MQC pathway.

## 2. FUNDC1 Structure

FUNDC1 is extensively expressed throughout the human body, especially in the heart [21]. In order to gain a deeper comprehension of the correlation between FUNDC1 and mitochondria, additional information regarding the structural components of FUNDC1 must be uncovered.

**Figure 1:**
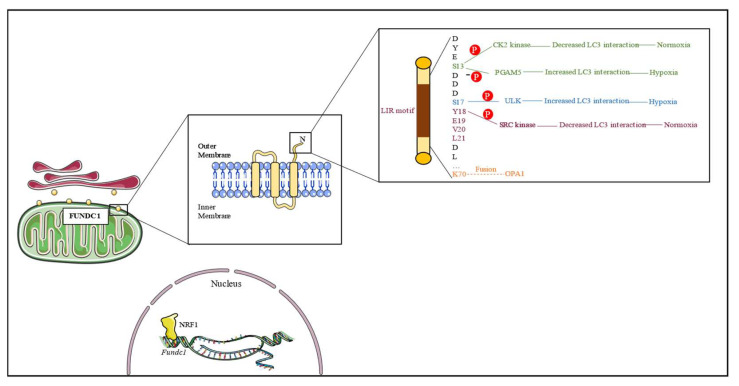
Overview of FUNDC1 structure. FUNDC1 is localized in the outer mitochondrial membrane (OMM) and mitochondrial-ER associated membranes (MAMs); FUNDC1 contains three transmembrane domains and its N-terminal is exposed to the cytoplasm, containing an LIR motif that facilitates LC3 interaction; In normoxia, CK2 and Src kinase would phosphorylate S13 and Y18 residue to reduce the interaction between FUNDC1 and LC3, whereas in hypoxia, PGAM5 dephosphorylates S13 residue and ULK phosphorylates S17 residue to increase the interaction between FUNDC1 and LC3; there is a mitochondrial fusion protein OPA1 binding site in the K70 residue; NRF1 transcriptionally regulates *Fundc1* expression.

FUNDC1 is composed of 155 amino acids, three transmembrane domains, and a typical LIR motif (Y18-E19-V20-L21) located near its N-terminus. This motif is exposed in the cytoplasm and facilitates the binding of LC3 to modulate mitophagy (Figure 1) [12]. Under hypoxic conditions, Src kinase becomes inactive, phosphorylation of Tyr18 is decreased, Ser13 is dephosphorylated by PGAM family member 5 (PGAM5), and Ser17 is phosphorylated by unc-51 like kinase 1 (ULK1), enhancing the interaction between FUNDC1 and LC3 and promoting mitophagy [12,22]. Scientists discovered that electrostatic repulsion is the primary cause of Tyr18 phosphorylation, which inhibits mitophagy [23]. Furthermore, the significance of phosphorylation of Tyr18 surpasses that of phosphorylation of Ser13 in regulating the binding affinity of LC3, indicating that Ser13 may have a supplementary function in the process of FUNDC1-mediated mitophagy [23]. In addition to its role in mitophagy, FUNDC1 has been demonstrated to be closely associated with mitochondrial dynamics and biogenesis. Therefore, a better understanding of its structural fundamentals would provide more direct evidence.

Studies have demonstrated that FUNDC1 and OPA1 collaborate to modulate mitochondrial dynamics [24]. Mutating lysine 70 (K70) of FUNDC1 eliminated its interaction with OPA1 and resulted in mitochondrial fragmentation, thereby indicating that FUNDC regulates mitochondrial morphology by binding to dynamic proteins [24]. Furthermore, the phosphorylation state of FUNDC1 also affects the binding affinity of dynamic proteins. Upon dephosphorylation during periods of stress, the interaction between FUNDC1 and OPA1 is reduced, while the association between FUNDC1 and dynamin-related protein 1 (DRP1) is augmented, thereby promoting mitochondrial fission and facilitating the onset of mitophagy [24]. In contrast, phosphorylation of FUNDC1 Ser13 results in a reduction of the interaction between DRP1 and FUNDC1, thereby inhibiting the process of FUNDC1-induced mitophagy [23]. Research has shown that mitophagy is conducive to mitochondrial biogenesis and necessitates the presence of FUNDC1 [25]. NRF1 is a transcription factor that can activate mitochondrial transcription factor A (TFAM) and increase mitochondrial biogenesis. Scientists discovered that NRF1 could also transcriptionally activate FUNDC1 by binding to the *Fundc1* promoter at −186/−176 [14].

In conclusion, it was discovered that FUNDC1 has a specific binding site with mitochondrial dynamic proteins, and various phosphorylation states of FUNDC1 mediated distinct mitochondrial morphology. In addition, the expression of FUNDC1 was regulated by the mitochondrial biogenesis activator NRF1 via its specific binding cites, and FUNDC1-induced mitophagy promoted mitochondrial biogenesis. In light of the structural relationship between FUNDC1, mitophagy, dynamics, and biogenesis, we propose that FUNDC1 is a regulator of the MQC system.

## 3. FUNDC1 and Mitochondrial Quality Control

### 3.1. FUNDC1 and Mitochondrial Biogenesis

Mitochondrial biogenesis is characterized by the generation of new mitochondria, an increase in mitochondrial content, and an elevation in the number of mitochondria, which stands in contrast to mitophagy. Mitochondria have their own genome; however, mtDNA codes for less than 1% of mitochondrial protein, which includes 13 essential components of the electron transport chain (ETC), as well as all rRNAs and tRNAs required for translation of the mtDNA-encoded proteins [26]; the remaining 99% of mitochondrial protein is coded by nuclear DNA (nDNA) [27]; thus, mitochondrial biogenesis and new protein synthesis require cooperation between the mitochondrial and nuclear genomes. Regulation of mitochondrial biogenesis is possible through mtDNA transcription and translation [28]. The PGC-1 protein family (PGC-1α, PGC1-β, and PGC-1) could activate mtDNA transcription [29], and PGC-1α, a transcriptional co-activator and pivotal regulator of mitochondrial biogenesis, can be activated through phosphorylation by AMPK or deacetylation by SIRT-1 [30]. The activation of PGC-1α initiates a cascade of nuclear transcription factors, including NRF-1, NRF-2, and ERR-α, culminating in the upregulation of TFAM, the ultimate mediator of mtDNA transcription and replication [31].

**Figure 2:**
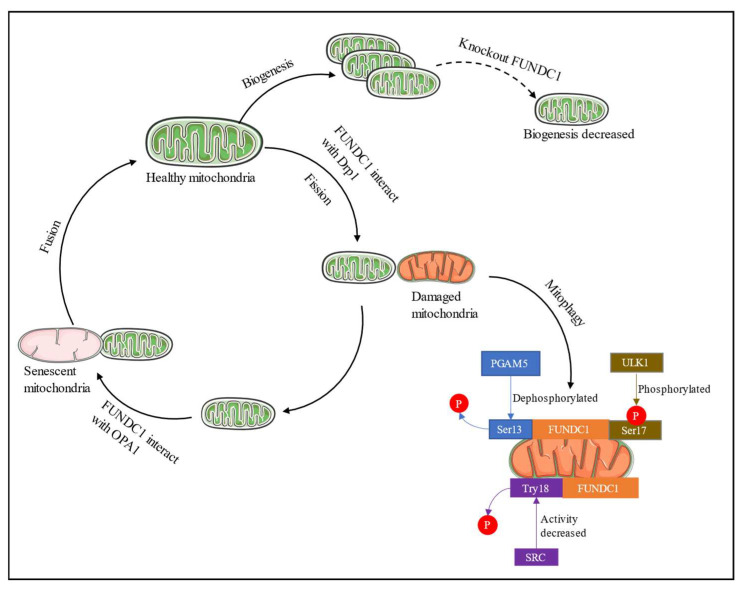
Overview of FUNDC1 regulate mitochondrial quality control (MQC) system. FUNDC1 initiates mitochondrial fission by interacting with DRP1 under stress; under normal conditions, it initiates mitochondrial fusion by interacting with OPA1. Fusion permitted two mitochondrial descendants and their content to mix within a mitochondrial population; FUDNC1 initiates mitophagy by dephosphorylating at Ser13, Try18, and phosphorylating at Ser17, respectively, by protein kinases such as PGAM5, ULK1, and Src kinases.

As the investigation of FUNDC1 progressed, scientists uncovered its association with mitochondrial biogenesis (Figure 2). The research findings indicate that the substantial reduction of PGC-1α levels occurred upon the KO of FUNDC1 in brown adipose tissue (BAT) [14]. FUNDC1-deficient BAT contained a relatively high proportion of mitochondria without cristae. When subjected to cold, the maximal oxygen consumption rates (OCRs) and mtDNA copy number of BAT lacking FUNDC1 did not exhibit a significant increase in comparison to the control group [14], indicating that FUNDC1 positively regulates mitochondrial biogenesis. Further investigation of this mechanism has revealed that FUNDC1 is controlled by PGC-1α/NRF1 [14]. KO FUNDC1 may have negatively influenced mitochondrial biogenesis. Nonetheless, a study has shown that the deficiency of FUNDC1 did not have any impact on the process of mitochondrial biogenesis. The deletion of FUNDC1 in cardiac progenitor cells (CPCs) had no effect on PGC-1α; even in FUNDC1 depletion cells, the proteins of mitochondrial respiratory chain complexes subunits remained elevated [32]. From the result mentioned above, the role of FUNDC1 in mitochondrial biogenesis appears to be ambiguous. Different cell lines may be responsible for the conflicting results. On the other hand, knockdown of FUNDC1 in CPC shows no effect on PGC-1α transcript level but increases the translation of this protein. Therefore, further research is needed to clarify the effect of FUNDC1 on mitochondrial biogenesis.

### 3.2. FUNDC1 and Mitochondrial Dynamics

Mitochondria are double-membraned, extremely dynamic organelles with a variety of morphologies to accomplish multiple functions. Controlling the variations in mitochondrial morphology are the opposing processes of fusion and fission. The process of mitochondrial fusion involves the fusion of the outer membrane followed by the fusion of the inner membrane, which is facilitated by the double membrane structure of mitochondria [33]. The process of mitochondrial fusion involves the merging of two distinct mitochondria to form a singular entity. This process has been found to augment oxidative phosphorylation [34], while also facilitating the transfer of mtDNA from damaged to healthy mitochondria [35]. However, there is a report indicating that the mingling of mtDNA genomes after fusion appears limited [36]; additional research is required. The process of mitochondrial fission, which is the antithesis of mitochondrial fusion, results in the division of a single mitochondrion into two smaller mitochondria, wherein one of the resultant mitochondria exhibits a diminished mitochondrial membrane potential (∆Ψm) [37]. Furthermore, fission directly correlates with mitophagy and, after mitochondrial fission, the lysosome will identify the fragment dysfunctional mitochondria with lower ∆Ψm and engulf it in an appropriate size [38]. Different morphology corresponds to various functions; consequently, the fine-tuning of mitochondrial dynamics is crucial for cellular health. The process of outer mitochondrial membrane (OMM) fusion in mammalian cells is facilitated by guanosine triphosphate hydrolases (GTPases) mitofusin (MFN) 1 and 2, which are situated in the OMM. Conversely, inner mitochondrial membrane (IMM) fusion is mediated by OPA1 [33]. In response to cellular signals, DRP1 undergoes translocation from the cytosol to mitochondria, where it oligomerizes and activates the OMM receptor [39] and associated proteins, including mitochondrial fission factor (MFF), mitochondrial dynamics protein 49/51 (MID49/51), and mitochondrial fission 1 protein (FIS1), ultimately driving mitochondrial fission [39]. Upon recruitment to the outer mitochondrial membrane (OMM), DRP1 assumes a ring-like configuration, thereby inducing constriction of the OMM and designating it as a potential site for future scission [40].

A recent discovery has revealed that FUNDC1 has the ability to regulate mitochondrial morphology. The suppression of FUNDC1 in HeLa cells elicits an increase in mitochondrial fusion, while the over expression of FUNDC1 impedes the process of fusion. The mechanism demonstrated that FUNDC1 and OPA1 can interact under normal conditions. FUNDC1 recruits DRP1 to mitochondria and promotes mitochondrial fission instead [24,41]. In contrast, the interaction between FUNDC1 and OPA1 decreases under stress conditions. FUNDC1 deficiency causes mitochondrial elongation in cardiomyocytes. FUNDC1 positively regulates FIS1 expression and functions as an upstream regulator of FIS1 [42]. Although FUNDC1 was initially thought to promote mitochondrial fusion, a distinct investigation revealed that its depletion in DU145 and LN229 cells led to an elevation in DRP1 phosphorylation and its translocation to mitochondria, indicating that FUNDC1 depletion induces mitochondrial fission rather than fusion [43]. The morphology of mitochondria is impacted by both the expression of FUNDC1 and its phosphorylation status. Upon phosphorylation of the S13 residue of FUNDC1, the association between FUNDC1 and DRP1 is diminished, while the association between FUNDC1 and OPA1 is augmented, resulting in mitochondrial elongation [23].

### 3.3. FUNDC1 and Mitophagy

Mitochondrial dysfunction results in programmed cell death and the production of reactive oxygen species (ROS), exacerbating cellular health and contributing to the development of severe conditions such as obesity and HDs. [44]. The elimination of dysfunctional mitochondria over a period of time is a crucial requirement for the proper functioning of cells and tissues. Mitophagy is a specialized form of autophagy that selectively targets dysfunctional mitochondria for degradation by autophagosomes/lysosomes [45], thereby maintaining the integrity of the mitochondrial network and function. At least two mitophagy regulation pathways have been identified: receptor-mediated and ubiquitin-mediated [46]. FUNDC1-induced mitophagy is one of the receptor-mediated mitophagy pathways that was discovered in 2012 [12].

The removal of FUNDC1 in white adipose tissue (WAT) leads to impaired mitophagy, accelerating the process of WAT remodeling and resulting in increased infiltration of adipose tissue-associated macrophages and chronic low-grade inflammation. This ultimately culminates in the development of severe obesity and insulin resistance when fed a high-fat diet (HFD) [47]. However, it is important to note that a lack of FUNDC1 does not have a universal impact on autophagy, thereby underscoring the necessity of mitophagy [12]. In addition, research indicates that multiple molecules enhance mitophagy via FUNDC1 pathway to maintain cell health. For instance, the administration of irisin enhances mitophagy in H9C2 cardiomyocytes, ameliorates glucose metabolism, and mitigates the oxidative stress induced by LPS stimulation [48]. Empagliflozin activates FUDNC1-dependent mitophagy, thereby reducing cardiac microvascular ischemia reperfusion (I/R) injury via the AMPK/ULK1 pathway [49]. However, FUNDC1-dependent mitophagy activation is not always advantageous for the cell. Pulmonary artery smooth muscle cells (PASMCs) from mice with hypoxic pulmonary hypertension (PH) exhibited a strong binding affinity between FUNDC1 and LC3B, resulting in increased ROS production and inhibited ubiquitination of hypoxia inducible factor 1 (HIF1), which causes severe hemodynamic changes and pulmonary vascular remodeling. With KO FUNDC1 in PASMCs, hypoxia-induced upregulation of ROS-HIF1 and PH was attenuated [50]. Further research is necessary to investigate the varying effects of mitophagy on human tissue in different diseases, as well as the role that FUNDC1 plays in this process. Subsequently, a detailed analysis will be presented regarding the impact of FUNDC1 on mitochondria and its influence on HDs.

## 4. FUNDC1, Mitochondrial Quality Control and Heart Diseases

### 4.1. Metabolic Cardiomyopathy

Cardiomyopathy is prevalent among individuals with obesity, insulin resistance (IR), and Type 2 diabetes (T2D) [51]. This type of cardiomyopathy, known as metabolic cardiomyopathy (MCM), is characterized by a similarly dysregulated metabolism in the development of cardiomyopathies and occurred independently of hypertension, coronary artery disease (CAD), or any risk factor for cardiovascular diseases [52,53]. In addition, researchers have discovered that many patients develop metabolic cardiomyopathy even in the absence of diabetes [54]; therefore, it is imperative to determine the molecular mechanisms underlying this pathological condition. It has been proposed that dysfunctional mitochondrial activation of aberrant cardiac metabolism plays a crucial role in mediating MCM. The elimination of damaged mitochondria through mitophagy plays a critical role in preventing additional harm to cardiomyocytes. Mitophagy impairment in ULK1-KO mice exacerbated cardiac diastolic and systolic dysfunction, whereas mitophagy upregulation restored cardiac function [55]. Under HFD, the majority of mitochondrial and dynamic function was lost, cardiac mitochondria fragmented, and the activity of citrate synthase and the electron transport chain (ETC) complexes decreased dramatically [56,57].

The expression of FUNDC1 was significantly increased in the cardiomyocytes of both diabetic rodents and humans (Table 1) [16]. FUNDC1 is accountable for the maintenance of MAMs formation, mitochondrial calcium homeostasis, mitophagy, and dynamic balance in MCM. The genetic downregulation of FUNDC1 impaired the connection between the ER and mitochondria, thereby impairing Ca^2+^ transfer from the ER to the mitochondria. FUNDC1 interacts with IP_3_R2 to facilitate Ca^2+^ transfer from a mechanistic standpoint. In the presence of heightened glucose levels, the activation of FUNDC1 results in the binding of CREB to the FIS1 promoter, thereby facilitating the formation of MAMs, augmenting the interaction between FUNDC1 and IP_3_R2, and inducing mitochondrial fragmentation. Consequently, the inhibition of FUNDC1 in cardiomyocytes affected by diabetes may lead to the restoration of mitochondrial function and the preservation of cardiac function. MAMs formation is essential for the MQC system, as demonstrated by previous research. Elevated glucose levels consistently led to the upregulation of MAMs formation. The overabundance of MAMs leads to excessive influx of ER Ca^2+^ to mitochondria, thereby triggering a pro-apoptotic signal [58]. This event results in a reduced mitochondrial biogenesis, downregulated the number of respiratory chain complexes, dynamic dysfunction and, ultimately, cellular death [16,59]. Salin and his colleagues hypothesized that ferulic acid and metformin are efficacious against MCM by reducing the formation of excessive MAMs and restoring mitochondrial function via a FUNDC1-related pathway [56]. Similar results were observed in muscle, where the absence of FUNDC1 protected against HFD-induced adiposity by enhancing insulin sensitivity and glucose tolerance [60]. Although the function of MAMs is not discussed in the original article, MAMs is essential for the maintenance of glucolipid metabolism

### 4.2. Cardiac Remodeling/Heart Failure

Cardiac remodeling can be divided into two categories: physiological and pathological. Physiological cardiac remodeling is a beneficial adaptation to various forms of stress, including exercise, growth, and pregnancy [61]. Conversely, pathological cardiac remodeling arises from cardiovascular stress, either internal or external, and may culminate in the development of heart failure (HF). To adapt to pressure-overloaded conditions such as hypertension, the heart undergoes concentric hypertrophy, where the cardiomyocytes typically increase in thickness [62]. This adaptation results in a reduction of ventricular wall stress and facilitates an increase in thickness of both the free wall and septum. However, despite the initial benefits of this adaptation, pathological cardiac remodeling can eventually lead to contractile dysfunction, which makes it difficult for the heart to pump enough oxygenated blood to support surrounding tissues, ultimately resulting in HF [63]. Although the molecular basis of cardiac remodeling and HF is still uncertain, accumulating evidence suggests that mitochondrial impairment plays a key role in their progression.

The expression of FUNDC1 was found to be significantly reduced in patients with heart failure, as opposed to those with diabetes [42]. In neonatal mouse cardiomyocytes, the KO of FUNDC1 was observed to impair mitochondrial function and cause severe cardiac damage. The abolition of FUNDC1 led to the destruction of MAMs formation, a marked decrease in the level of Ca^2+^ in mitochondria ([Ca^2+^]_mito_) and cytosol, the suppression of FIS1, and the elongation of mitochondria [42]. FUNDC1 interacts with calnexin accumulated at MAMs, the association between FUNDC1 and calnexin decreased during stress, and FUNDC1 interacts with DRP1 instead [13]. This study highlights the significant role of MAMs in protein interaction and suggests that pathological disruption of MAM formation can result in a dysfunctional cellular response. Oleanolic acid (OA) has been shown to have a favorable effect during cardiac remodeling, which would be disrupted in the absence of FUNDC1 [64]. Similarly, alpha-lipoic acid (α-LA) has been found to reduce pressure overload-induced left ventricular hypertrophy and dysfunction through the FUNDC1 pathway [65].

### 4.3. Myocardial Ischemia-Reperfusion Injury

Smoking, drinking, insufficient physical activity, and other unhealthy behaviors may increase the risk of myocardial infarction (MI). When acute MI has occurred, it is crucial to restore coronary blood flow promptly, since the extent of myocardial injury is directly proportional to the ischemic duration. Revascularization treatments can cause cardiomyocyte mortality and myocardial damage, which is referred to as ischemia/reperfusion (I/R) injury. During the reperfusion phase, ROS and [Ca^2+^]_mito_ accumulation occur, leading to decreased ∆Ψm and the opening of the mitochondrial permeability transition pore (mPTP). The principal outcome of cardiac I/R injury is the induction of oxidative stress in cardiomyocytes, which arises from substantial perturbations in mitochondrial metabolism [66]. Given the critical role of mitochondrial quality changes in I/R injuries, regulating mitochondrial balance appropriately could be a potential therapeutic target for cardiac I/R injury.

**Table 1:** Summary of FUNDC1 in MCM, cardiac remodeling/HF, and cardiac I/R injury.

Author (Year)	Diseases	Tissue	Function of FUNDC1
Shengnan Wu (2019) [16]	Metabolic Disorder	Cardiac	1. FUNDC1 increased in diabetes heart 2. MAMs formation increased 3. Interaction between FUNDC1 and Ip_3_r2 increased, mitochondrial Ca^2+^ increased 4. cardiac mitochondria impaired due to the upregulation of FUNDC1
Salin Raj (2022) [59]	Metabolic Disorder	Cardiac	1. FUNDC1 increased in diabetes heart 2. MAMs formation increased 3. Ferulic acid alleviate cardiac mitochondrial dysfunction through decrease FUNDC1
Tingting Fu (2018) [60]	Metabolic Disorder	Skeletal Muscle	1. Lack of FUNDC1 improved systemic insulin sensitivity and glucose tolerance
Shengnan Wu (2017) [42]	Heart Failure	Cardiac	1. FUNDC1 decreased in patients with heart failure 2. MAMs formation decreased 3. Interaction between FUNDC1 and Ip_3_r2 increased, mitochondrial Ca^2+^ increased 4. FUNDC1 deficient cause severe cardiac dysfunction
Yan Gong (2022) [64]	Cardiac Remodeling	Cardiac	1. Oleanolic acid (OA) alleviated aging-induced changes in myocardial remodeling 2. FUNDC1 deficient nullified OA benefit effect
Wenjia Li (2020) [65]	Heart Failure	Cardiac	1. Alpha-lipoic acid (α-LA) reduce cardiac injury through FUDNC1-dependent pathway
Hao Zhou (2018) [67]	I/R injury	Cardiac	1. FUNDC1 was inactivity during I/R injury 2. I/R injury leads to insufficient FUNDC1 induced-mitophagy
Chen Cai (2022) [49]	I/R injury	Cardiac	1. Empagliflozin alleviate cardiac I/R injury by activating FUNDC1

Numerous studies have demonstrated that the inhibition of FUNDC1-mediated mitophagy during cardiac I/R injury may result in heightened cardiac injury [67]. The impairment of FUNDC1-induced mitophagy results in the accumulation of damaged mitochondria, which exacerbates cardiac infarction by accelerating cell apoptosis. Researchers have determined that MAMs can modulate cellular function, which is related to I/R injury. Several MAMs proteins serve a protective function against I/R injury. For instance CLIC4, a MAM CL^−^ channel, resides within MAMs. The ablation of CLIC4 activates the RyR2 channel, which is the Ca^2+^ channel for cardiomyocytes and ultimately leads to [Ca^2+^]_mito_ overload, increasing ROS production, decreasing ∆Ψm and enhancing infarction upon cardiac I/R injury [68]. NOX4, another MAMs protein, was considered a pro-survival mechanism that restricts infarct size by promoting Akt-dependent phosphorylation of IP_3_R, thereby inhibiting calcium flux and mPTP-dependent necrosis [69]. Moreover, Empagliflozin has been shown to reduce cardiac microvascular I/R injury by protecting mitochondrial function in a FUNDC1-related manner [49]. In light of the relationship between FUNDC1 and MAMs, the disruption of MAMs by a decrease in FUNDC1 may result in severe myocardial injury in I/R, because the “protective protein” in MAMs cannot function properly without the hub. FUNDC1 would also interact with other MAMs proteins to facilitate Ca^2+^ transfer; thus, we must determine FUNDC1’s dual role upon I/R injury. Unfortunately, there has been no experimentation conducted to ascertain the protective or detrimental effects of FUNDC1 on cardiomyocytes. Consequently, further investigation is necessary in this area.

## 5. Exercise Improves Cardiac Function Might through AMPK/FUNDC1

Extensive evidence has shown that physical activity is beneficial for the prevention and treatment of heart-related diseases. Mitochondria are abundant in cardiac tissue, which meets the body’s energy needs. However, mitochondrial dysregulation significantly contributes to cardiac pathology. Exercise may provide benefits to some mitochondrial regions. For instance, MG53, a myokine secreted after exercise, can preserve mitochondrial integrity and prevent further damage to the heart following an I/R injury by binding to cardiolipin (CL) [70]. Irisin, another myokine, also protects cardiac tissue against I/R-induced injury [71]. Irisin is a disintegrin and metalloproteinase (ADAM) family enzyme that acts on the integrin V5/Akt and is the mitophagy activator induced by FUNDC1 [48]. Cardiomyocyte apoptosis and ROS production were inhibited by Irisin-FUNDC1 when H9C2 cardiomyocytes were damaged by LPS [48]. Emerging evidence confirms that mitochondria play a crucial role in the cardiac benefits of exercise. Increasing mitochondrial biogenesis and mitophagy in response to exercise is one of the “beneficial” mechanisms. In rodents with MI, a 4-week treadmill exercise activated the SIRT1/PGC-1α/PI3K/Akt pathway, initiating mitochondrial biogenesis, reducing cardiac fibrosis and oxidative stress [72]. Resistance exercise has positive effects on the heart. After 12 weeks of ladder climbing five days per week, PGC-1α and TFAM expression was increased in cardiac mitochondria, accompanied by decreased proton leakage and ROS production, which contributes to the maintenance of diabetic cardiac contractility [73]. The purpose of the exercise was not only to increase mitochondrial biogenesis, but also to improve the clearance of damaged mitochondria. According to studies, endurance exercise substantially increased mitophagy flux and protected the heart from doxorubicin (DOX)-induced damage [74]. Research suggests that mitophagy serves a protective function in heart injury, and exercise is among the activators of this process. Specifically, physically active mice exhibited more sustained mitophagy and appropriate mitochondrial quality control [74]. FUNDC1 could be one of the potential targets, although the precise mechanism of improving mitochondrial function is still being debated.

**Figure 3:**
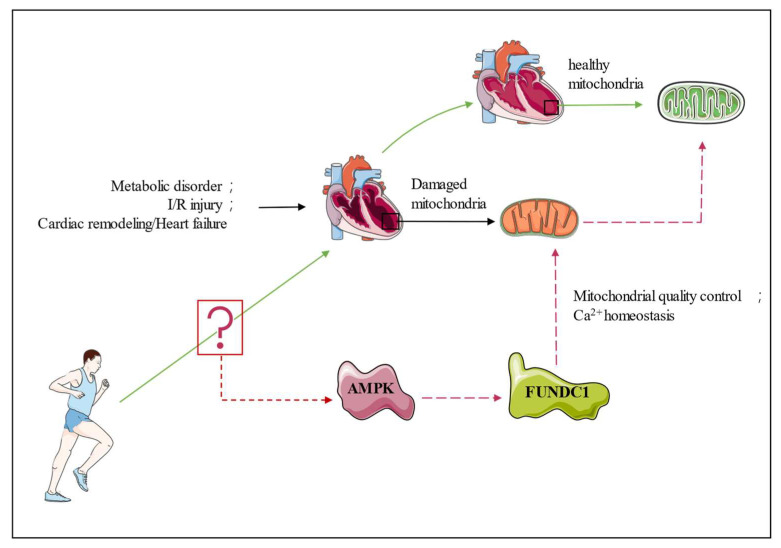
Overview of exercise’s beneficial role for the heart through the AMPK/FUNDC1 pathway. Exercise may increase AMPK activity and its expression in cardiomyocytes, leading to a modification of FUNDC1. FUDNC1 may modulate the MCQ system and [Ca^2+^]_mito_ homeostasis in order to enhance cardiac mitochondrial function and ultimately promote cardiac function.

Currently, there is a paucity of research that directly examines the association between physical exercise and FUNDC1. Liang Yu and colleagues (2020) conducted a study which revealed that a four-week training program led to an upsurge in the expression of FUNDC1 in skeletal muscle [75]. The level of FUNDC1 was observed to be dependent on exercise intensity, with high-intensity exercise inducing a higher level of FUNDC1 compared to moderate-intensity exercise [75]. Furthermore, studies indicate that the upregulation of FUNDC1 induced by exercise may be mediated through the AMPK/ULK1 signaling pathways [76]. AMPK is a serine/threonine kinase that is conserved throughout evolution and was first identified as a key regulator of energy homeostasis [77]. Exercise is considered to be one of the most effective activators of AMPK owing to its impact on energy metabolism, and it is also a potential pathway that benefits cardiac function [78]. Mice with STZ/HFD-induced diabetic cardiomyopathy were subjected to a 16-week treadmill running program at a speed of 10 m/min for 1 h per day. The findings indicate that physical activity had a notable impact on the quantity of respiratory chain complexes and ∆Ψm levels, ameliorated systolic dysfunction, and safeguarded the heart against ROS accumulation by means of an AMPK-dependent mechanism [79]. Significantly greater phosphorylation and total AMPK protein expression are observed in the exercise diabetes group compared to the non-exercise diabetes group [79]. The activation of downstream signaling molecules, such as PGC-1α [80], ULK1 [81], SIRT1 [82], and FUNDC1 [83], by AMPK, could result in mitochondrial adaptation. Due to the relationship between exercise and AMPK, AICAR, the agonist of AMPK, was always regarded as the exercise mimetic during in vitro experiments [84]. The investigation of AMPK and FUNDC1 revealed evidence implicating AMPK/FUNDC1 in the pathophysiology of cardiovascular diseases (Figure 3). AMPK could stimulate FUNDC1 expression [76]. This promotion may be the factor that protects the I/R myocardium. In the I/R murine model, the expression of PLK1 was significantly reduced, and overexpressing PLK1 in H9c2 cells increased the survival rate following hypoxia and reoxygenation treatment. PLK1 also activated the expression of phosphorylation AMPK (P-AMPK) in H9c2 cells, leading an increase in FUNDC1 expression. Inhibition of FUNDC1 expression leads to the inhibition of mitophagy and subsequently nullifies all beneficial effects, suggesting that AMPK/FUNDC1 may play a crucial role in maintaining mitochondrial homeostasis and preserving cardiac function during I/R injury [83]. However, another study found that decreased AMPK activity promotes FUNDC1 upregulation in neonatal diabetic mouse cardiomyocytes, as well as increased MAMs formation and [Ca^2+^]_mito_ level, resulting in cardiac dysfunction [16]

FUNDC1 may be beneficial or detrimental to the heart. FUNDC1 could initiate mitophagy to eliminate damaged mitochondria, thereby protecting against I/R injury, cardiac remodeling, and heart failure. While the heart was under high glucose condition, the increment of FUNDC1 accelerated mitochondria damage by upregulating MAMs formation and [Ca^2+^]_mito_ level. From the evidence we have mentioned earlier, it is reasonable to state that exercise enhances cardiac function through the AMPK/FUNDC1 pathway. Exercise serves as an activator of AMPK, thereby augmenting AMPK expression and phosphorylation, which in turn exerts an impact on FUNDC1. Although the available evidence is limited, it suggests that these alterations confer a favorable influence on cardiac function.

## 6. Conclusions

FUNDC1, a mitophagy regulated protein that locates in OMM and MAMs, regulates both the MQC system and the formation of MAMs. Numerous pieces of evidence indicated that FUNDC1 is closely related to HDs. In this review, we examined the molecular structure of FUNDC1 and outlined its cellular function in the MQC system, highlighting its protective role in cardiac remodeling/heart failure and cardiac I/R injury. However, overexpression of FUNDC1 appears to exacerbate metabolic cardiomyopathy. In conclusion, we also postulate that exercise may improve cardiac function via the AMPK/FUNDC1 pathway. The underlying mechanisms of FUNDC1 in HDs, particularly its dual role in distinct types of HDs, remain unclear.

MAMs have been identified as a unique “organelle” that regulates cellular processes, including ER stress, MQC, inflammation, Ca^2+^ transduction, and lipid synthesis [85]. FUNDC1 is responsible for maintaining the integrity of MAMs and the homeostasis of mitochondrial Ca^2+^ by interacting with IP_3_R2 [16,42]. Ca^2+^ may explain why FUNDC1 played distinct roles in various types of HDs. Cardiac [Ca^2+^]_mito_ is a promising factor for inducing cardiac contraction; during intense cardiac workload, the concentration of [Ca^2+^]_mito_ increased to stimulate pyruvate dehydrogenase activity [86] and the oxidation rate of nicotinamide adenine dinucleotide hydrogen (NADH), thereby increasing ATP production. Given that the [Ca^2+^]_mito_ influx increased four to five times from that of the basal rate, it is plausible that a [Ca^2+^]_mito_ deficiency would ruin the heart’s energy production [87]. Nonetheless, excessive [Ca^2+^]_mito_ causes cardiomyocyte damage and cell mortality by opening of the mPTP [88]. mPTP opens in the IMM, allowing unrestricted access of molecules 1.5 kDa, including protons, resulting in uncoupling of oxidative phosphorylation, ATP depletion, and cell death [89]. This may explain why Ca^2+^ is a double-edged sword for cardiovascular health. In addition, the FUNDC1 deficiency-induced collapse of MAMs has an effect on the MQC process, including abnormal mitochondrial morphology regulation, insufficient biogenesis, and mitophagy, suggesting that the FUNDC1/MAMs/mitochondria axis may be the primary target of HDs.

Exercising improves cardiac function by activating signaling molecules and the transcriptional network. AMPK is highly responsive to exercise and facilitates advantageous cellular adaptations in the cardiac muscle [78]. In contrast, AMPK regulates FUNDC1 expression in cardiac tissue [16,76,83]. On the basis of the evidence presented above, we hypothesize that exercise improves cardiac function through the AMPK/FUNDC1 pathway. However, there is scant evidence linking exercise and AMPK/FUDNC1.

There are several unanswered concerns regarding the therapeutic potential of FUNDC1 in HDs. First, the regulatory function and interaction of FUNDC1 with the MAMs protein are unknown. Second, elucidating the regulation mechanism and specific function of Ca^2+^ for mitochondria under the modification of FUNDC1/MAMs in various types of HDs is crucial. Due to the limitations of in vitro cell experiments and animal experiments, it is necessary to conduct human tissue experiments. Fourth, the optimal excise mode (type, intensity, and duration) for various HDs remains undetermined. It is imperative to clarify the extent of FUNDC1’s involvement in maintaining mitochondrial homeostasis and cellular function. This is anticipated to be the prospective therapeutic strategy in the future.

## Data Availability

Not applicable.
